# Supplementary material for: Modeling cardiac fibroblast heterogeneity from human pluripotent stem cell-derived epicardial cells
Source: Nat Commun. 2023 Dec 11;14:8183. doi: 10.1038/s41467-023-43312-0 (PMC10713677; doi:10.1038/s41467-023-43312-0)
Supplement: Supplementary file 1 — Supplementary Information [file 41467_2023_43312_MOESM1_ESM.pdf]

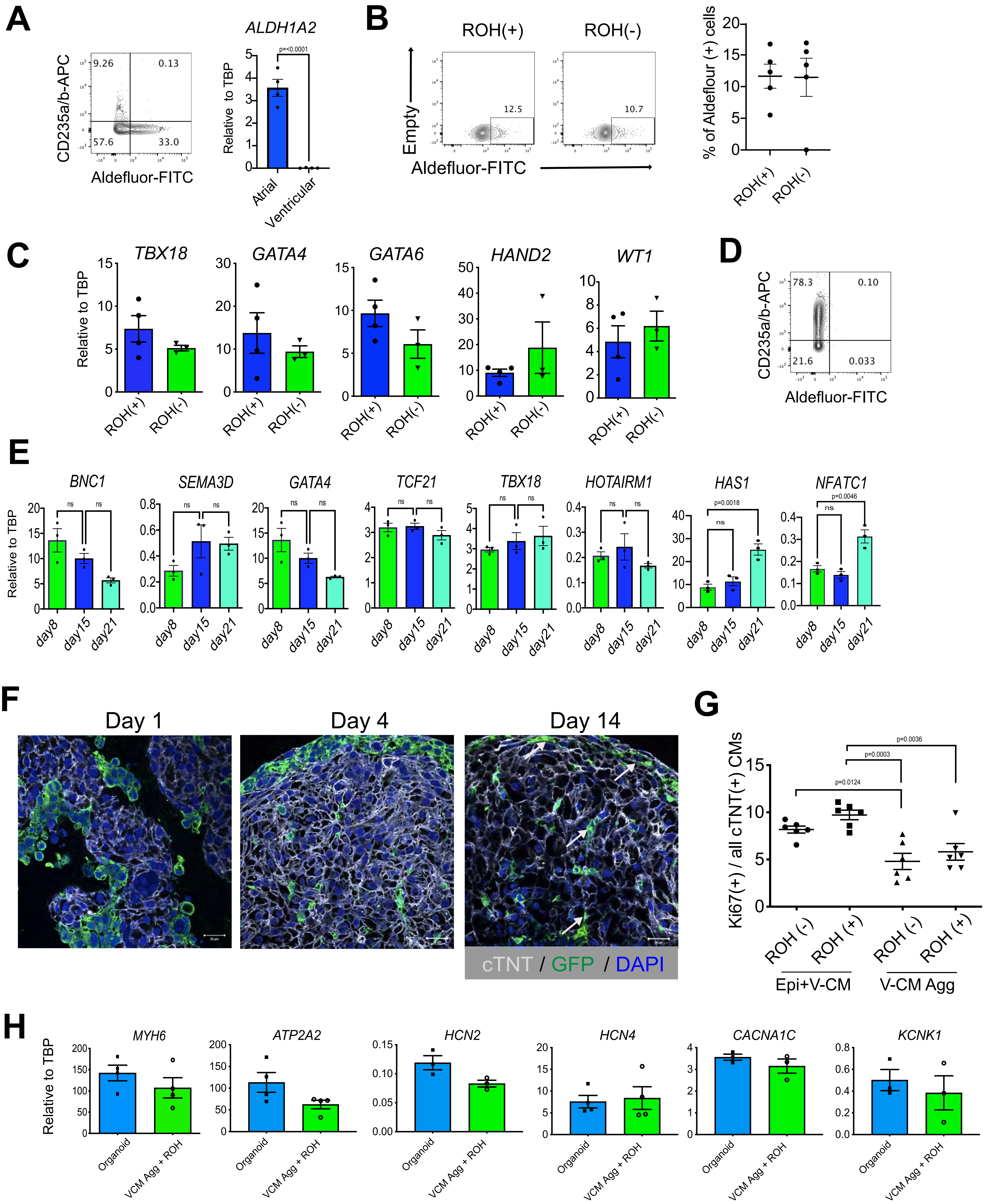

A
CD235a/b-APC
Aldefluor-FITC
9.26
0.13
57.6
33.0
ALDH1A2
Relative to TBP
p=<0.0001
Atrial
Ventricular
B
ROH(+)
ROH(-)
Empty
Aldefluor-FITC
12.5
10.7
% of Aldeflour (+) cells
C
TBX18
GATA4
GATA6
HAND2
WT1
Relative to TBP
D
78.3
0.10
21.6
0.033
E
BNC1
SEMA3D
GATA4
TCF21
TBX18
HOTAIRM1
HAS1
NFATC1
ns
p=0.0018
p=0.0046
day8
day15
day21
F
Day 1
Day 4
Day 14
cTNT / GFP / DAPI
G
Ki67(+) / all cTNT(+) CMs
p=0.0036
p=0.0003
p=0.0124
ROH (-)
ROH (+)
Epi+V-CM
V-CM Agg
H
MYH6
ATP2A2
HCN2
HCN4
CACNA1C
KCNK1
Organoid
VCM Agg + ROH

Supplementary Figure 1. Generation of cardiac organoids with ventricular cardiomyocytes and epicardial cells. (A) Left; Representative flow cytometry analyses of Aldefluor and CD235a/b in day 4 atrial mesoderm. Right; RT-qPCR expression analyses of *ALDH1A2* in atrial and ventricular mesoderm (N=4 biologically independent samples). (B) Left; Representative flow cytometry analysis of Aldefluor in ROH-treated and untreated epicardial cells at day 8. Right; Quantification of the percentage of Aldefluor-positive cells in the indicated conditions (N=5 biologically independent samples). (C) RT-qPCR expression analyses of epicardial-related and cardiac transcription factors (*TBX18, GATA4, GATA6, HAND2, WT1*) in the ROH-treated and untreated (ROH (-)) epicardial cells (N=4 biologically independent samples). (D) Representative flow cytometry analyses of Aldefluor and CD235a/b in day 4 ventricular mesoderm. (E) RT-qPCR expression analyses of epicardial-related genes (*BNC1, TBX18, GATA4, SEMA3D, TCF21, HOTAIRM1, HAS1, NFATC*) in the epicardial cells at the indicated time of culture (N=3 biologically independent samples). (F) Representative immunostaining of GFP and cTNT in day1, 4, 14 cardiac organoids. White arrows indicate elongated GFP-positive EPDCs. GFP; epi-derived cells (EPDCs), Scale bar; 20um. (G) Quantification of the changes in the percentage of Ki67-positive cardiomyocytes in the cardiac organoids and cardiomyocyte aggregates (VCM Agg) treated as indicated (N=6 biologically independent samples). (H) RT-qPCR expression analyses of sarcomere-related (*MYH6*), Ca^{2+} handling-related (*ATP2A2*) and ion channel-related genes (*HCN2, HCN4, CACNA1C, KCNK1*) in the indicated conditions (N=4 biologically independent samples). Statistical analysis was performed by two-sided unpaired t-test (A) and by one-way ANOVA with Tukey's multiple comparisons (E) and (G). All error bars represent SEM.

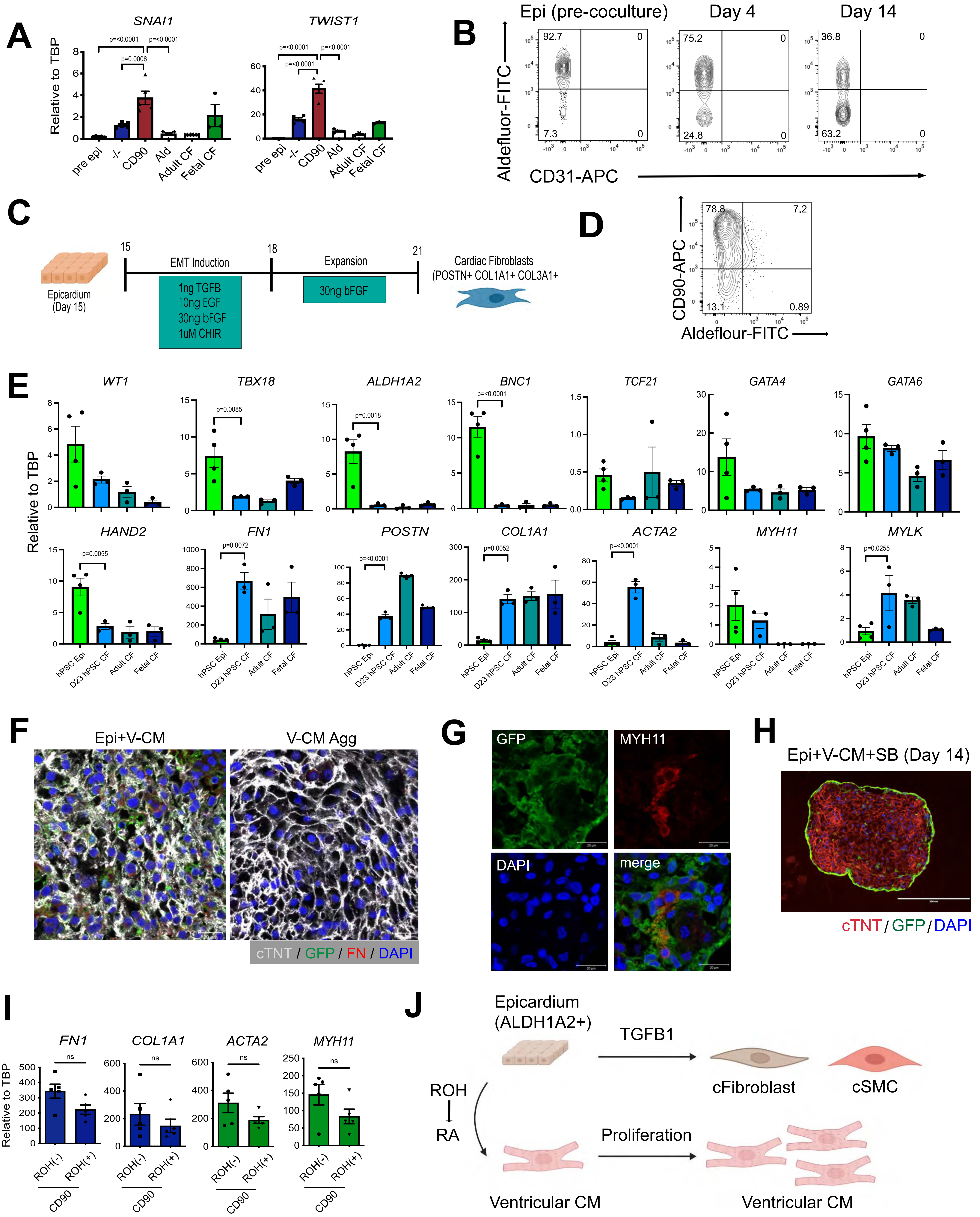

A
SNAI1
TWIST1
Relative to TBP
pre epi
-/-
CD90
Ald
Adult CF
Fetal CF
B
Epi (pre-coculture)
Day 4
Day 14
Aldefluor-FITC
CD31-APC
C
Epicardium (Day 15)
EMT Induction
Expansion
1ng TGFB
10ng EGF
30ng bFGF
1uM CHIR
30ng bFGF
Cardiac Fibroblasts (POSTN+ COL1A1+ COL3A1+
D
CD90-APC
Aldefluor-FITC
E
WT1
TBX18
ALDH1A2
BNC1
TCF21
GATA4
GATA6
HAND2
FN1
POSTN
COL1A1
ACTA2
MYH11
MYLK
hPSC Epi
D23 hPSC CF
Adult CF
Fetal CF
F
Epi+V-CM
V-CM Agg
cTNT / GFP / FN / DAPI
G
GFP
MYH11
DAPI
merge
H
Epi+V-CM+SB (Day 14)
cTNT / GFP / DAPI
I
FN1
COL1A1
ACTA2
MYH11
Relative to TBP
ROH(-)
ROH(+)
CD90
J
Epicardium (ALDH1A2+)
TGFB1
cFibroblast
cSMC
ROH
RA
Proliferation
Ventricular CM
Ventricular CM

Supplementary Figure 2. Characterization epicardial-derived cells (EPDCs) in the cardiac organoids. (A) RT-qPCR expression analyses of EMT-related (*SNAI1*, *TWIST1*) genes in the indicated populations (N=5 biologically independent samples). Adult and fetal CF was included as a reference (N=3 biologically independent samples each). Epi: day15 epicardium prior to coculture. (B) Representative flow cytometry analysis of Aldefluor and CD31 in EPDCs in the cardiac organoids in the indicated time points. (C) Protocol to directly differentiate hPSC-derived epicardial cells into cardiac fibroblasts. (D) Representative flow cytometry analysis of CD90 and Aldefluor in directly differentiated CFs derived from epicardial cells. (E) RT-qPCR expression analyses of epicardial (*WT1, TBX18, ALDH1A2*, *BNC1*), transcription factor (*TCF21, GATA4, GATA6, HAND2)*, extracellular matrix (*FN1, POSTN, COL1A1*), and smooth muscle (*ACTA2, MYH11, MYLK*) genes in the indicated populations (N=3 biologically independent samples). Adult and fetal CF were included as a reference (N=3 biologically independent samples each). Epi: day15 epicardium, (F) Representative immunostaining of fibronectin (FN) and GFP (EPDCs) in the cardiac organoids and ventricular cardiomyocyte aggregates (V-CM Agg). Scale bar; 20um. (G) Representative immunostaining of MYH11 in the GFP-positive EPDCs in the cardiac organoids. Scale bar; 20um. (H) Representative immunostaining of day 14 SB-treated cardiac organoids. Scale bar; 200um. (I) RT-qPCR expression analyses of *FN1*, *COL1, ACTA2,* and *MYH11* in CD90-positive populations in the ROH-treated and untreated organoids (N=5 biologically independent samples). (J) Schematic of the cardiac organoids showing the interaction between the cardiomyocytes and the epicardial cells. Statistical analysis was performed by two-sided unpaired t-test. Created with BioRender.com

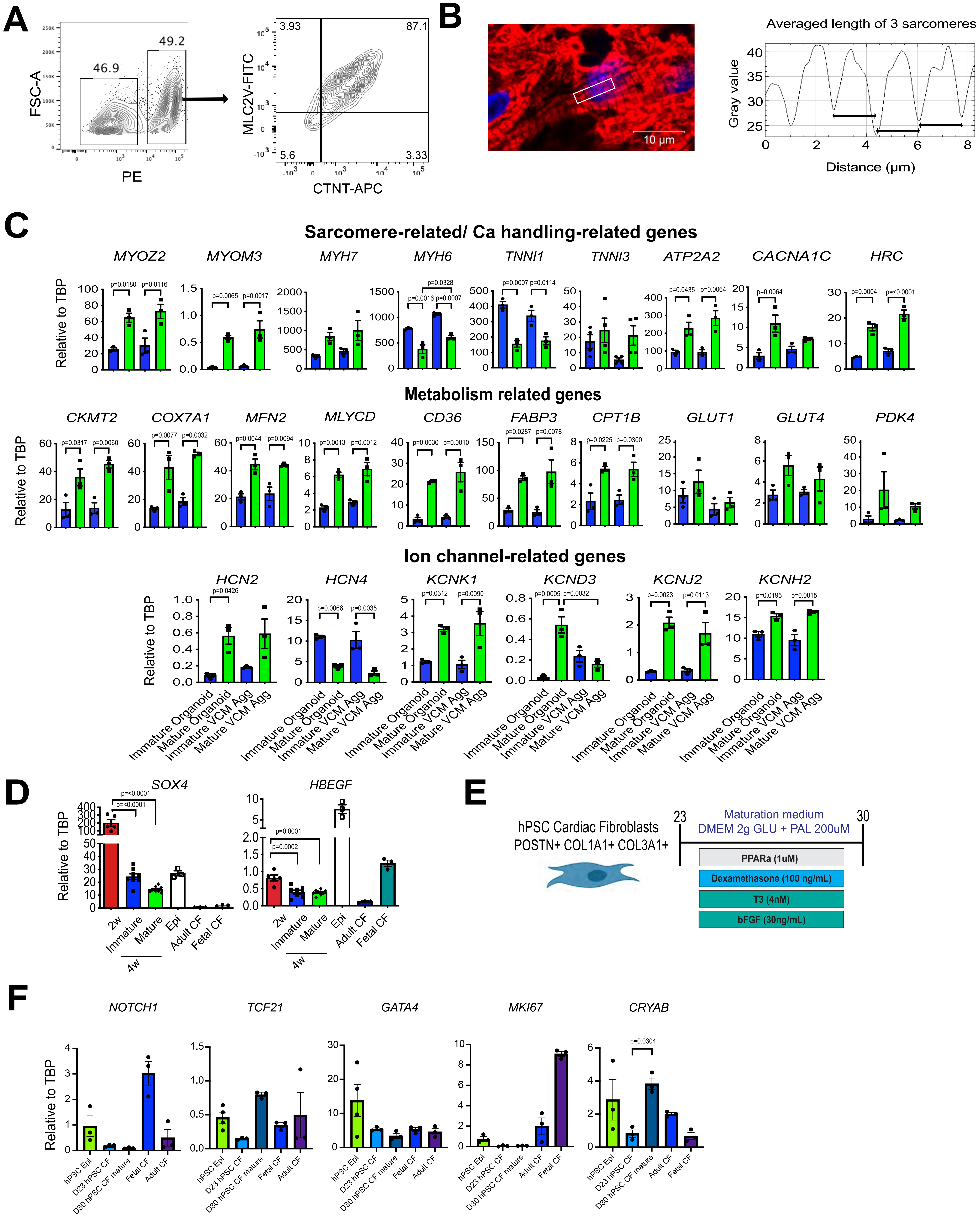
A
FSC-A
PE
46.9
49.2
MLC2V-FITC
CTNT-APC
3.93
87.1
5.6
3.33
B
10 µm
Averaged length of 3 sarcomeres
Gray value
Distance (µm)
C
Sarcomere-related/ Ca handling-related genes
MYOZ2
MYOM3
MYH7
MYH6
TNNI1
TNNI3
ATP2A2
CACNA1C
HRC
Relative to TBP
p=0.0180
p=0.0116
p=0.0065
p=0.0017
p=0.0328
p=0.0016
p=0.0007
p=0.0007
p=0.0114
p=0.0435
p=0.0064
p=0.0064
p=0.0004
p=<0.0001
Metabolism related genes
CKMT2
COX7A1
MFN2
MLYCD
CD36
FABP3
CPT1B
GLUT1
GLUT4
PDK4
p=0.0317
p=0.0060
p=0.0077
p=0.0032
p=0.0044
p=0.0094
p=0.0013
p=0.0012
p=0.0030
p=0.0010
p=0.0287
p=0.0078
p=0.0225
p=0.0300
Ion channel-related genes
HCN2
HCN4
KCNK1
KCND3
KCNJ2
KCNH2
p=0.0426
p=0.0066
p=0.0035
p=0.0312
p=0.0090
p=0.0005
p=0.0032
p=0.0023
p=0.0113
p=0.0195
p=0.0015
Immature Organoid
Mature Organoid
Immature VCM Agg
Mature VCM Agg
D
SOX4
HBEGF
p=<0.0001
p=<0.0001
p=0.0001
p=0.0002
2w
Immature
Mature
Epi
Adult CF
Fetal CF
4w
E
hPSC Cardiac Fibroblasts
POSTN+ COL1A1+ COL3A1+
23
30
Maturation medium
DMEM 2g GLU + PAL 200uM
PPARa (1uM)
Dexamethasone (100 ng/mL)
T3 (4nM)
bFGF (30ng/mL)
F
NOTCH1
TCF21
GATA4
MKI67
CRYAB
p=0.0304
hPSC Epi
D23 hPSC CF
D30 hPSC CF mature
Fetal CF
Adult CF

Supplementary Figure 3. Metabolic maturation in cardiac organoids. (A) Representative flow cytometry analysis of cTNT and MLC2v in RFP$^+$ sorted population in the cardiac organoids. (B) The method to measure sarcomere length in an image of cTNT staining. Sarcomere length was measured in an area with a white square by Image J. Scale bar; 10um. (C) RT-qPCR expression analyses of the sarcomere (*MYOZ2, MYOM3, DES, MYH7, MYH6, TNNI1, TNNI3*), Ca handling (*ATP2A2, HRC*), fatty acid oxidation (*CD36, FABP3, CPT1B, MLYCD*) and mitochondria (*CKMT2, COX7A1, MFN2*), Ion channel (*KCNK1, HCN2, KCND3, CACNA1C, KCNJ2, KCNH2, HCN4*) -related genes in FACS isolated cardiomyocyte from the immature and mature cardiac organoids and immature and mature ventricular cardiomyocyte aggregates (VCM Agg) (N=3 biologically independent samples). (D) RT-qPCR expression analyses of *SOX4* and *HBEGF* in FACS isolated CD90$^+$ cardiac fibroblasts populations from the immature and mature cardiac organoids (N=4 biologically independent samples). Adult and fetal CF was included as a reference (N=3 biologically independent samples each). epi: day15 epicardial cells prior to the initiation of coculture (N=3 biologically independent samples), CF: cardiac fibroblasts. (E) Protocol to culture directly differentiate hPSC-derived epicardial cells in the maturation media. (F) RT-qPCR expression analyses of indicated genes in the day23 CFs and day30 CFs cultured in the maturation media (N=3). Adult and fetal CF were included as a reference (N=3 biologically independent samples). epi: day15 epicardial cells (N=3 biologically independent samples), Statistical analysis was performed by one-way ANOVA with Tukey's multiple comparisons in (C) (D) (F). All error bars represent SEM. Created with BioRender.com

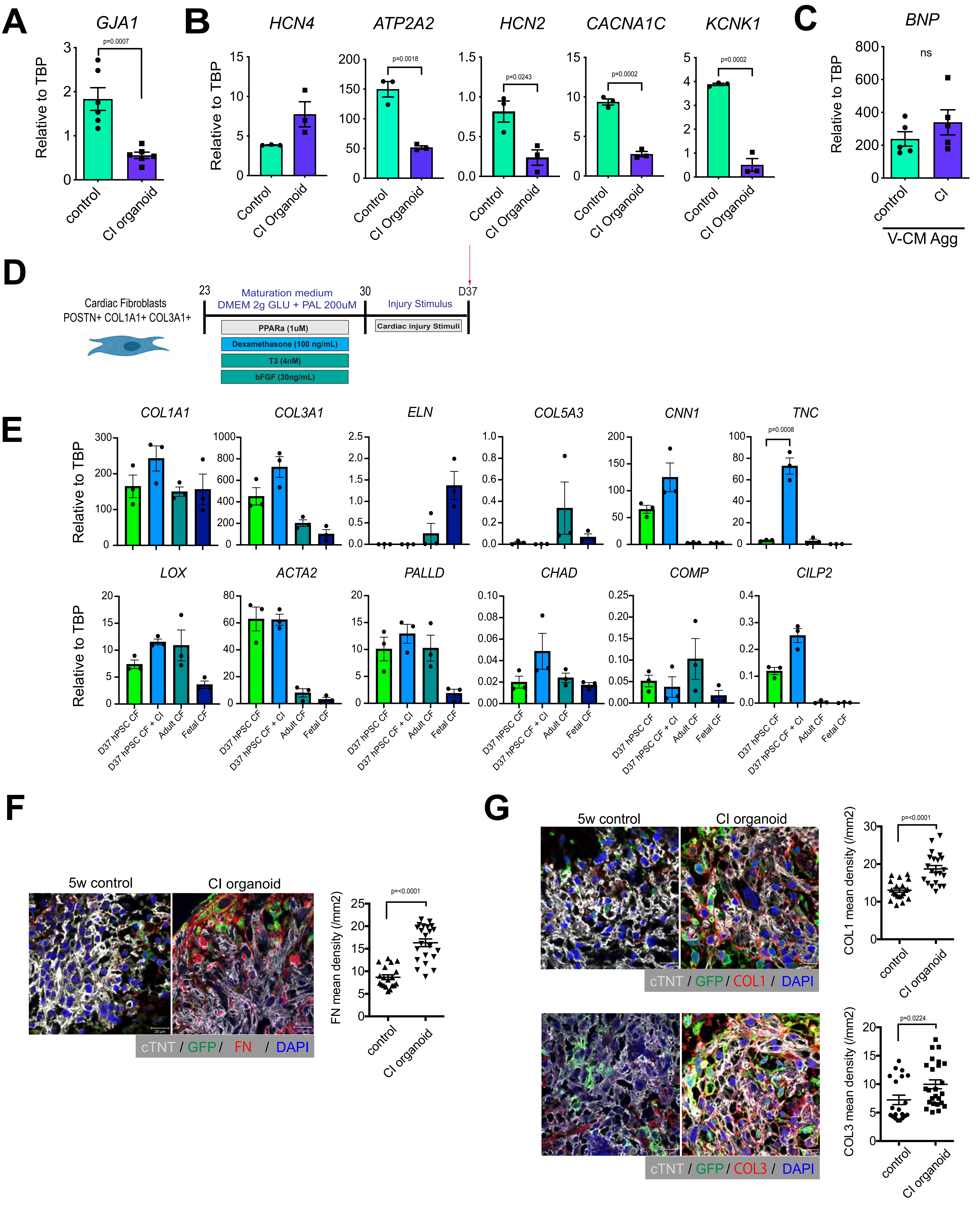

A
GJA1
p=0.0007
Relative to TBP
control
CI organoid
B
HCN4
ATP2A2
HCN2
CACNA1C
KCNK1
p=0.0018
p=0.0243
p=0.0002
p=0.0002
Control
CI Organoid
C
BNP
ns
CI
V-CM Agg
D
Cardiac Fibroblasts
POSTN+ COL1A1+ COL3A1+
23
Maturation medium
DMEM 2g GLU + PAL 200uM
30
Injury Stimulus
D37
PPARa (1uM)
Dexamethasone (100 ng/mL)
T3 (4nM)
bFGF (30ng/mL)
Cardiac injury Stimuli
E
COL1A1
COL3A1
ELN
COL5A3
CNN1
TNC
p=0.0008
LOX
ACTA2
PALLD
CHAD
COMP
CILP2
D37 hPSC CF
D37 hPSC CF + CI
Adult CF
Fetal CF
F
5w control
CI organoid
cTNT / GFP / FN / DAPI
FN mean density (/mm2)
p=<0.0001
G
cTNT / GFP / COL1 / DAPI
COL1 mean density (/mm2)
cTNT / GFP / COL3 / DAPI
COL3 mean density (/mm2)
p=0.0224

Supplementary Figure 4. Modeling cardiac injury in cardiac organoids. (A) RT-qPCR expression analyses of *GJA1* in the FACS isolated cardiomyocyte populations from the control and CI organoids (N=6 biologically independent samples). (B) RT-qPCR expression analyses of Ca^{2+} handling-related (*ATP2A2*) and ion channel-related (*HCN4, HCN2, CACNA1C, KCNK1*) in the FACS isolated cardiomyocyte populations from the control and CI organoids (N=3 biologically independent samples). (C) RT-qPCR expression analyses of heart failure marker (*BNP*) in the ventricular cardiomyocyte aggregates (V-CM-Agg) cultured as indicated (N=5 biologically independent samples). (D) Left; Representative immunostaining of fibronectin (FN) in the control and CI organoids at 5 weeks of culture. Scale bar; 20um. Right; Quantification of the FN density in the two organoid populations (N=19 biologically independent samples). (E) Upper left; Representative immunostaining of collagen type 1 (COL1) in the control and CI organoids at 5 weeks of culture. Scale bar; 20um. Upper right; Quantification of the COL1 density in the two organoid populations (N=20 biologically independent samples). Lower left; Representative immunostaining of collagen type 3 (COL3) in the control and CI organoids at 5 weeks of culture. Scale bar; 20um. Lower right; Quantification of the COL3 density in two organoid populations (N=20 biologically independent samples). (F) Protocol to expose the cardiac injury stimuli in directly differentiated CFs derived from hPSC-epicardial cells. (G) RT-qPCR expression analyses of extracellular matrix (*COL1A1, COL3A1*), cytoskeleton (*ACTA2, PALLD, CNN1*), fibrosis-related (*TNC, LOX*), and matrifibrocyte-related (*CHAD, COMP, CILP2*) genes in the indicated populations (N=3 biologically independent samples for matrifibrocyte genes, N=6 biologically independent samples for others). Adult and fetal CF RNA was included as a reference (N=3 biologically independent samples each). CI organoid: cardiac injury organoid. CF: cardiac fibroblasts. Statistical analysis was performed by two-sided unpaired t-test. All error bars represent SEM. Created with BioRender.com

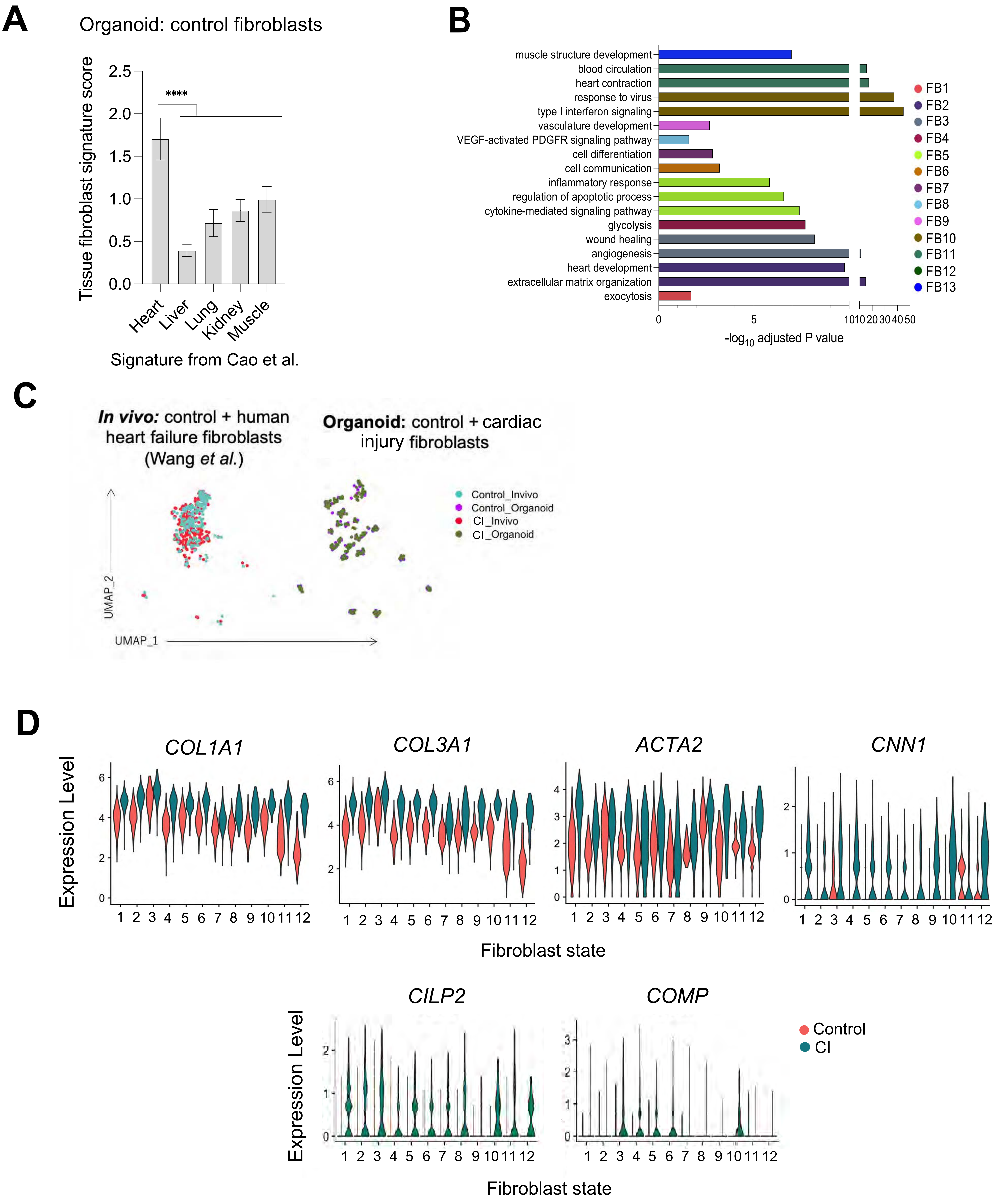
A
Organoid: control fibroblasts
Tissue fibroblast signature score
2.5
2.0
1.5
1.0
0.5
0.0
****
Heart
Liver
Lung
Kidney
Muscle
Signature from Cao et al.
B
muscle structure development
blood circulation
heart contraction
response to virus
type I interferon signaling
vasculature development
VEGF-activated PDGFR signaling pathway
cell differentiation
cell communication
inflammatory response
regulation of apoptotic process
cytokine-mediated signaling pathway
glycolysis
wound healing
angiogenesis
heart development
extracellular matrix organization
exocytosis
0
5
10
10
20
30
40
50
-log10 adjusted P value
FB1
FB2
FB3
FB4
FB5
FB6
FB7
FB8
FB9
FB10
FB11
FB12
FB13
C
In vivo: control + human heart failure fibroblasts (Wang et al.)
Organoid: control + cardiac injury fibroblasts
Control_Invivo
Control_Organoid
CI_Invivo
CI_Organoid
UMAP_2
UMAP_1
D
COL1A1
COL3A1
ACTA2
CNN1
Expression Level
Fibroblast state
1 2 3 4 5 6 7 8 9 10 11 12
CILP2
COMP
Control
CI

Supplementary Figure 5. Single-cell RNA sequencing analyses of the cardiac organoids. (A) Flow chart of single-cell RNA sequencing sample workflow. RFP-positive and GFP-positive fractions of control and CI organoids were sorted and labelled individually using lipid-tagged antibodies, and subsequently pooled for sequencing using the 10x Genomics platform and MULTI-seq. Following sequencing, the data was demultiplexed to assign cells back to RFP or GFP fractions. (B) UMAP dimensionality reduction of total cells in control and CI samples split by RFP and GFP fractions. (C) UMAP plots showing expression of smooth muscle/pericyte and fibroblast-related genes (*RGS5, PDGFRB, ACTN1, CALD1, TPM2, COL1A1, COL3A1, FN1, POSTN, THY1*). (D) UMAP plots showing expression of adipocyte-related genes (*PPARG, KLF5, BMP2, CEBPA, CD24, DGAT2*). (E) Pathway enrichment analysis was performed for each cardiomyocyte cluster in Figure 5 (gProfiler, GO: Biological Processes). The top unique pathways in each cluster are displayed with their enrichment score ($-\log_{10}$ of adjusted p-value). (F) Violin plots showing expression of *NPPA* and *NPPB* in each cardiomyocyte cluster in control and CI samples. (G) Integration analysis of cardiomyocyte population in control and CI organoid samples with cardiomyocytes from *in vivo* human coronary heart failure dataset (Wang *et al.*). Datasets were downsampled to 400 cells each to avoid biases associated with differential cell numbers. CM: cardiomyocyte, CI organoid: cardiac injury organoid.

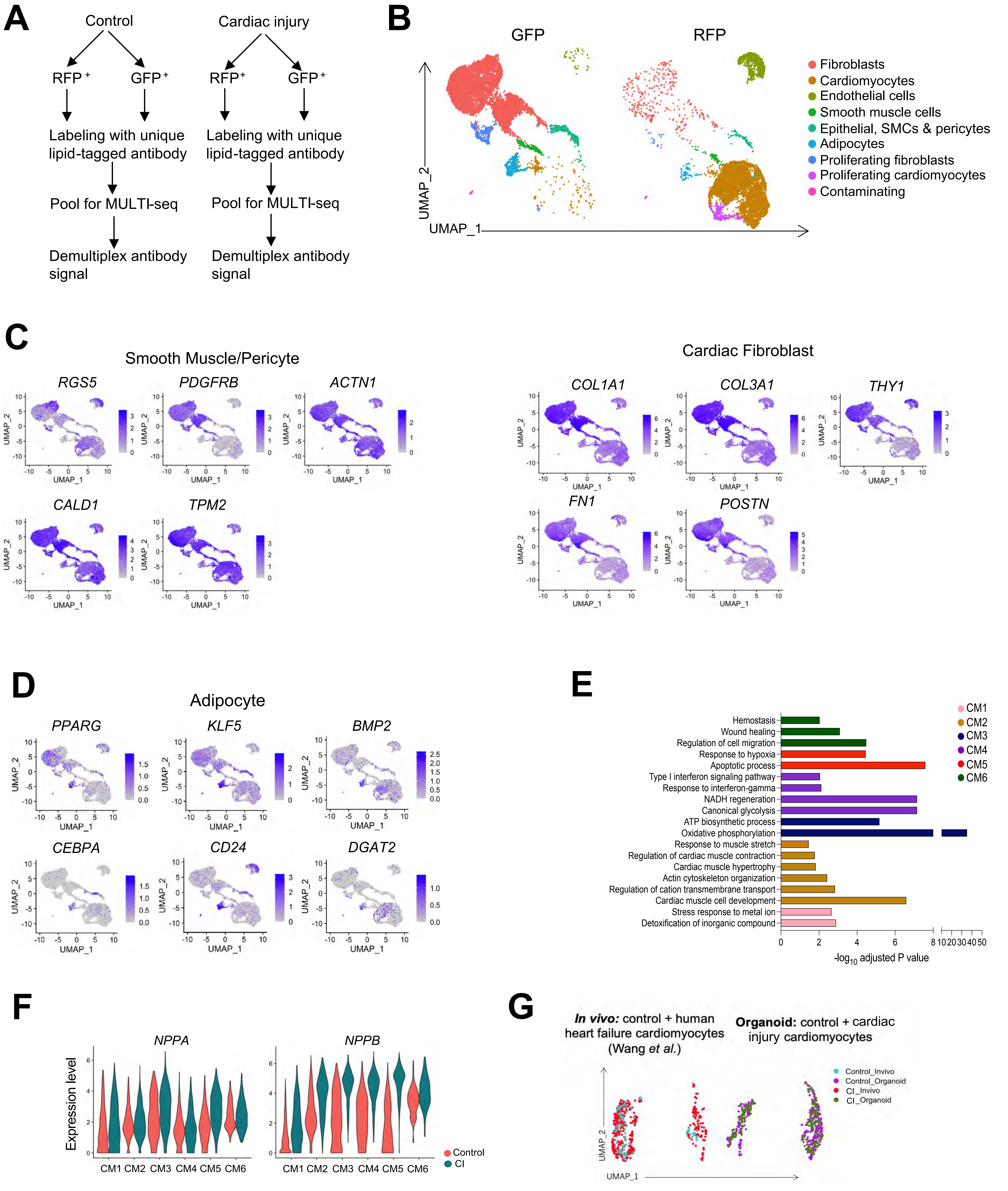
A
Control
RFP+
GFP+
Labeling with unique lipid-tagged antibody
Pool for MULTI-seq
Demultiplex antibody signal
Cardiac injury
RFP+
GFP+
Labeling with unique lipid-tagged antibody
Pool for MULTI-seq
Demultiplex antibody signal
B
GFP
RFP
UMAP_2
UMAP_1
Fibroblasts
Cardiomyocytes
Endothelial cells
Smooth muscle cells
Epithelial, SMCs & pericytes
Adipocytes
Proliferating fibroblasts
Proliferating cardiomyocytes
Contaminating
C
Smooth Muscle/Pericyte
RGS5
PDGFRB
ACTN1
CALD1
TPM2
Cardiac Fibroblast
COL1A1
COL3A1
THY1
FN1
POSTN
D
Adipocyte
PPARG
KLF5
BMP2
CEBPA
CD24
DGAT2
E
Hemostasis
Wound healing
Regulation of cell migration
Response to hypoxia
Apoptotic process
Type I interferon signaling pathway
Response to interferon-gamma
NADH regeneration
Canonical glycolysis
ATP biosynthetic process
Oxidative phosphorylation
Response to muscle stretch
Regulation of cardiac muscle contraction
Cardiac muscle hypertrophy
Actin cytoskeleton organization
Regulation of cation transmembrane transport
Cardiac muscle cell development
Stress response to metal ion
Detoxification of inorganic compound
CM1
CM2
CM3
CM4
CM5
CM6
-log10 adjusted P value
F
NPPA
NPPB
Expression level
CM1 CM2 CM3 CM4 CM5 CM6
Control
CI
G
In vivo: control + human heart failure cardiomyocytes (Wang et al.)
Organoid: control + cardiac injury cardiomyocytes
Control_Invivo
Control_Organoid
CI_Invivo
CI_Organoid
UMAP_2
UMAP_1

Supplementary Figure 6. Molecular characterization of fibroblasts in the cardiac organoids. (A) Single-cell RNA sequence data of human fetal fibroblasts from 5 tissues (Cao *et al.*) were compared to identify tissue-specific fibroblast signatures. Each tissue-specific fibroblast signature was used to score control organoid fibroblasts. One-way ANOVA was performed for each tissue signature compared to the heart signature. (B) Pathway enrichment analysis was performed for each fibroblast cluster in Figure 6 (gProfiler, GO: Biological Processes). The top unique pathways in each cluster are displayed with their enrichment score (-$\log_{10}$ of adjusted p-value). (C) Integration analysis of fibroblast population in control and CI organoid samples with fibroblasts from *in vivo* human coronary heart failure dataset (Wang *et al.*). Datasets were downsampled to 400 cells each to avoid biases associated with differential cell numbers. (D) Violin plots showing expression of fibrosis-related genes (*COL1A1, COL3A1, ACTA2, CNN1, CILP2, COMP*) in each fibroblast cluster in control and CI samples. FB: fibroblast, CI organoid: cardiac injury organoid.

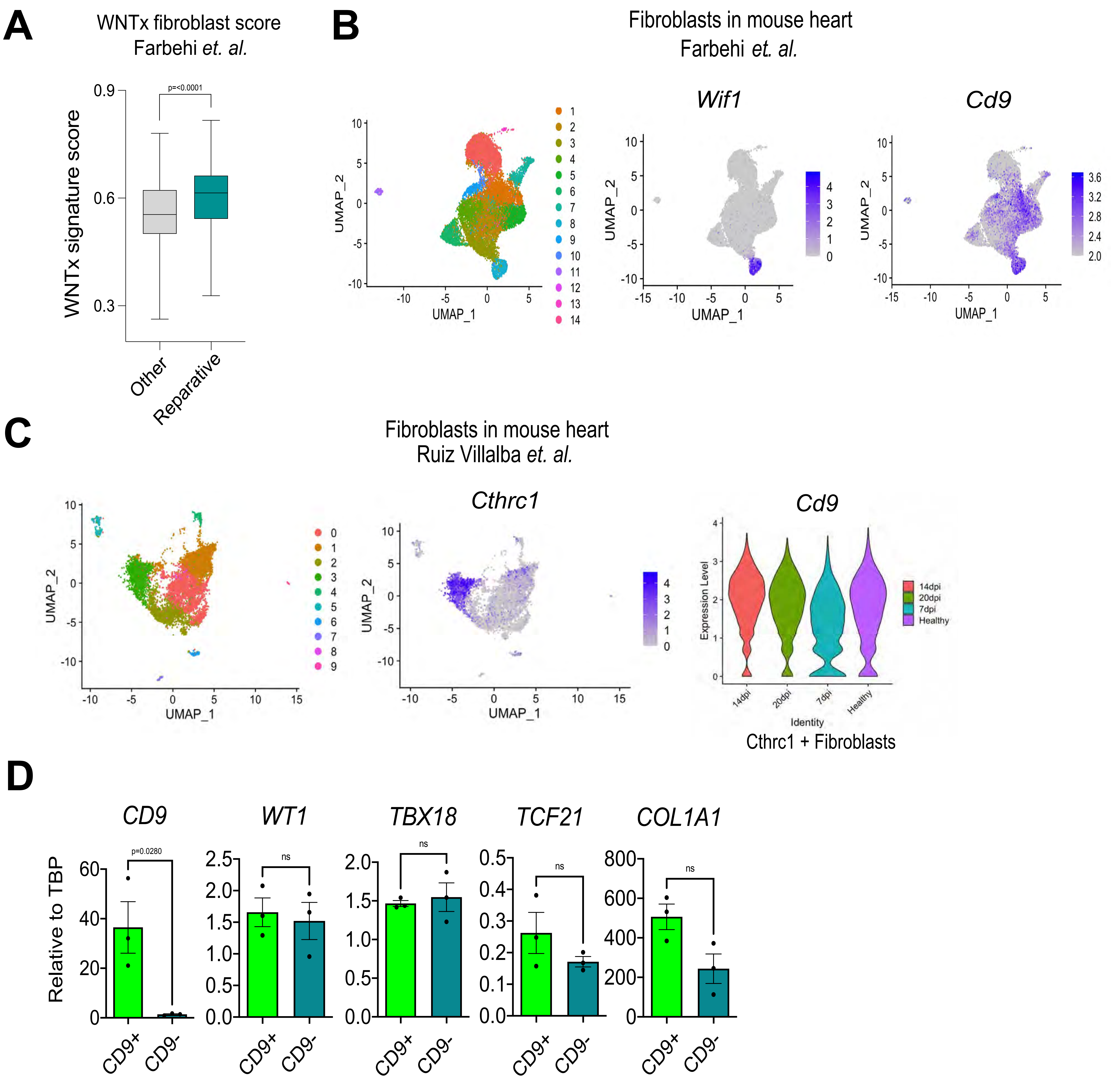

A
WNTx fibroblast score
Farbehi et. al.
WNTx signature score
p=<0.0001
Other
Reparative
B
Fibroblasts in mouse heart
Farbehi et. al.
Wif1
Cd9
UMAP_1
UMAP_2
C
Fibroblasts in mouse heart
Ruiz Villalba et. al.
Cthrc1
Cd9
14dpi
20dpi
7dpi
Healthy
Expression Level
Identity
Cthrc1 + Fibroblasts
D
CD9
WT1
TBX18
TCF21
COL1A1
Relative to TBP
p=0.0280
ns
CD9+
CD9-

Supplementary Figure 7. Identification of a CD9$^+$ reparative like fibroblast population (A) A signature for the WNTx fibroblast cluster defined by Farbehi *et al*. was generated by comparing the WTNx cluster to all other fibroblasts. "Reparative" and "other" fibroblasts from the CI organoids (see Figure 7) were scored with this signature. An unpaired t-test was performed to compare the two groups. (B) Clustering analysis of fibroblast populations and UMAP plots showing expression of *Wif1* and *Cd9* in mouse heart *in vivo* (Farberi *et al*.) (C) Left; Clustering analysis of fibroblast populations and UMAP plots showing expression of *Cthrc1*. Right; Violin plots showing expression of *Cd9* in Cthrc1-positive fibroblasts at the indicated time points. All data are analyzed from the mouse heart dataset of Ruiz Villalba *et al*. (D) RT-qPCR expression analyses of indicated genes in the CD9$^+$ and CD9$^-$ cardiac fibroblast populations isolated from the CI organoids (N=3 biologically independent samples). CI organoid: cardiac injury organoid. Statistical analysis was performed by two-sided unpaired t-test.

A

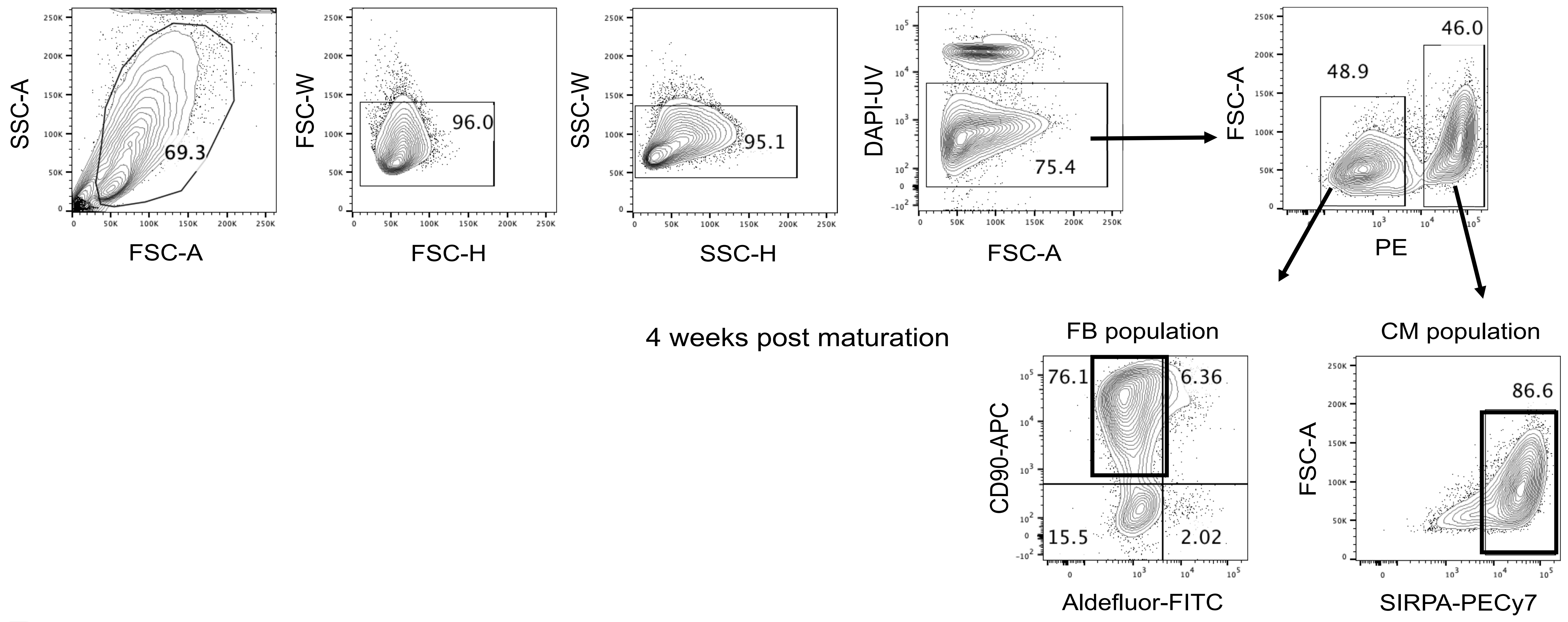

SSC-A
FSC-A
69.3
FSC-W
FSC-H
96.0
SSC-W
SSC-H
95.1
DAPI-UV
FSC-A
75.4
FSC-A
PE
48.9
46.0
4 weeks post maturation
FB population
CD90-APC
Aldefluor-FITC
76.1
6.36
15.5
2.02
CM population
FSC-A
SIRPA-PECy7
86.6

B

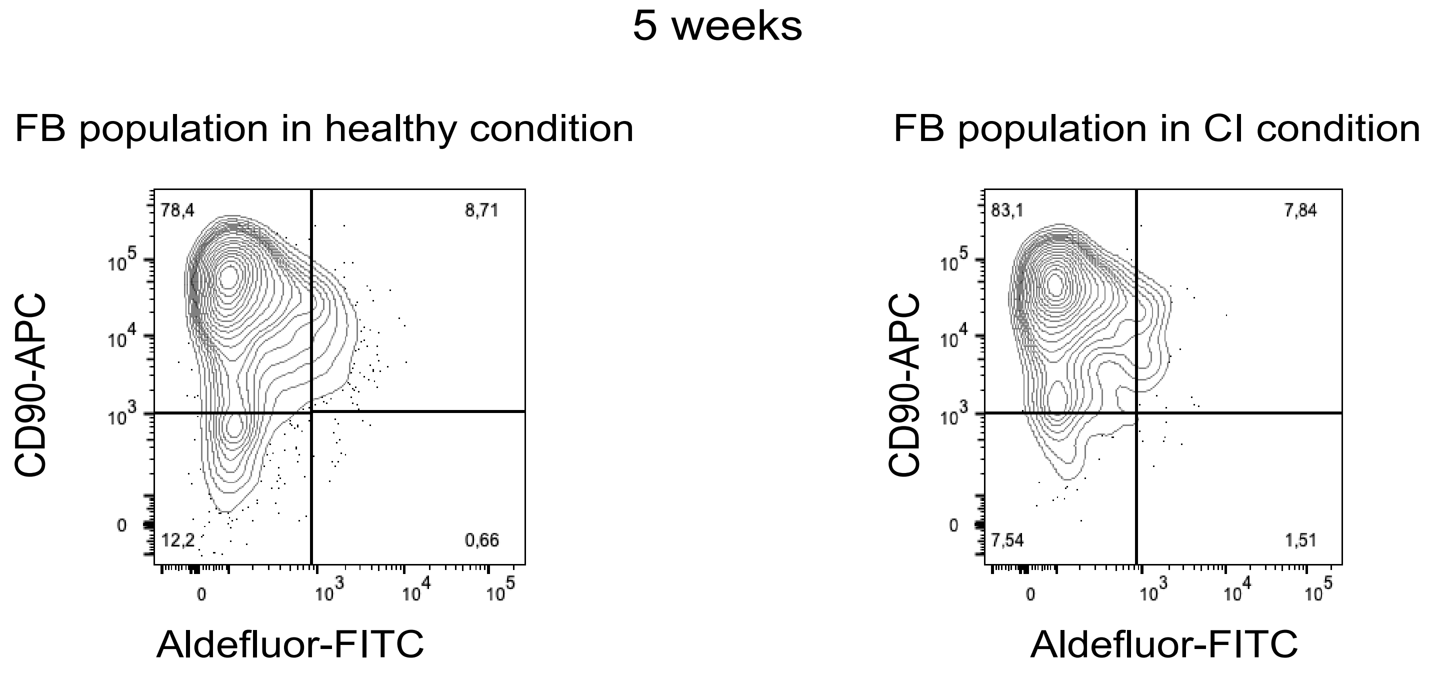

5 weeks
FB population in healthy condition
CD90-APC
Aldefluor-FITC
78,4
8,71
12,2
0,66
FB population in CI condition
CD90-APC
Aldefluor-FITC
83,1
7,84
7,54
1,51

C

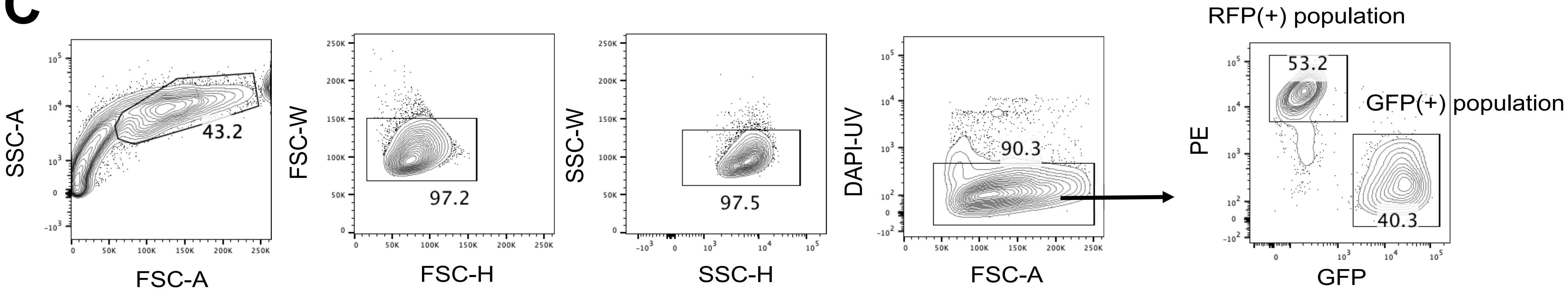

SSC-A
FSC-A
43.2
FSC-W
FSC-H
97.2
SSC-W
SSC-H
97.5
DAPI-UV
FSC-A
90.3
RFP(+) population
GFP(+) population
PE
GFP
53.2
40.3

Supplementary Figure 8. Gating strategy for flow-cytometric analysis. (A) Gating strategy for sorting RFP(+) / SIRPA(+)-cardiomyocytes and CD90(+)-fibroblasts derived from epicardial cells in the cardiac organoid used in Figure 2-4 and Supplementary Figure 2-4. Representative flow cytometry result of CD90 and Aldefluor in EPDC population in the 4-week cardiac organoids are shown. (B) Representative flow cytometry analysis of CD90 and Aldefluor in EPDC population in the 5-week cardiac organoids with or without cardiac injury. (C) Gating strategy for sorting RFP-positive and GFP-positive fractions of the cardiac organoids for MULTI-seq shown in Figure 5, 6 and Supplementary Figure 5, 6.
