# Supplementary material for: Modeling cardiac fibroblast heterogeneity from human pluripotent stem cell-derived epicardial cells
Source: Nat Commun. 2023 Dec 11;14:8183. doi: 10.1038/s41467-023-43312-0 (PMC10713677; doi:10.1038/s41467-023-43312-0)
Supplement: Supplementary file 3 — Description of Additional Supplementary Files [file 41467_2023_43312_MOESM3_ESM.pdf]

Description of Additional Supplementary Files:

Supplementary Data 1: Differentially expressed genes in each cell type in the combined analysis of all cells in control and heart failure organoids.

Supplementary Data 2: Differentially expressed genes in CM6 heart failure vs control

Supplementary Data 3: Differentially expressed genes induced in HF vs control

Supplementary Data 4: Primer lists for RT-qPCR.
