# Supplementary material for: Modeling cardiac fibroblast heterogeneity from human pluripotent stem cell-derived epicardial cells
Source: Nat Commun. 2023 Dec 11;14:8183. doi: 10.1038/s41467-023-43312-0 (PMC10713677; doi:10.1038/s41467-023-43312-0)
Supplement: Supplementary file 8 — Reporting Summary [file 41467_2023_43312_MOESM8_ESM.pdf]

nature portfolio

Corresponding author(s): Gordon Keller & Shunsuke Funakoshi

Last updated by author(s): Oct 22, 2023

Reporting Summary

Nature Portfolio wishes to improve the reproducibility of the work that we publish. This form provides structure for consistency and transparency in reporting. For further information on Nature Portfolio policies, see our Editorial Policies and the Editorial Policy Checklist.

Statistics

For all statistical analyses, confirm that the following items are present in the figure legend, table legend, main text, or Methods section.

n/a	Confirmed	
☐	☒	The exact sample size (*n*) for each experimental group/condition, given as a discrete number and unit of measurement
☐	☒	A statement on whether measurements were taken from distinct samples or whether the same sample was measured repeatedly
☐	☒	The statistical test(s) used AND whether they are one- or two-sided<br>*Only common tests should be described solely by name; describe more complex techniques in the Methods section.*
☒	☐	A description of all covariates tested
☒	☐	A description of any assumptions or corrections, such as tests of normality and adjustment for multiple comparisons
☐	☒	A full description of the statistical parameters including central tendency (e.g. means) or other basic estimates (e.g. regression coefficient) AND variation (e.g. standard deviation) or associated estimates of uncertainty (e.g. confidence intervals)
☐	☒	For null hypothesis testing, the test statistic (e.g. F, t, r) with confidence intervals, effect sizes, degrees of freedom and P value noted<br>*Give P values as exact values whenever suitable.*
☒	☐	For Bayesian analysis, information on the choice of priors and Markov chain Monte Carlo settings
☒	☐	For hierarchical and complex designs, identification of the appropriate level for tests and full reporting of outcomes
☒	☐	Estimates of effect sizes (e.g. Cohen's d, Pearson's r), indicating how they were calculated

Our web collection on statistics for biologists contains articles on many of the points above.

Software and code

Policy information about availability of computer code

Data collection	Zen Blue 2.3 software (Carl Zeiss), Zen Black 2.3 software (Carl Zeiss), Seahorse XF24 analyzer and Wave software (2.6.1) (Agilent)<br>FACS DIVA (8), CFX384 Touch real-time PCR detection system (Biorad), MetaMorph software (7.10)(Molecular devices), Zeiss LSM700<br>scRNAseq; 10X library prep chemistry was Chromium Single Cell 3'v3, sequenced on Illumina Nova seq 6000.
Data analysis	Images were analyzed using Zen Blue 3.5 software (Carl Zeiss), Zen Black 3.0 software (Carl Zeiss), and ImageJ (Fiji version) Software (1.52) (NIH).<br>Graph and statistical analysis were generated using GraphPad Prism 9.0 (GraphPad Software).<br>Flow cytometric data were analyzed using FlowJo 10.7 software (Tree Star).<br>Seahorse data were analyzed using Seahorse Wave software (2.6.1) (Agilent).<br>scRNAseq analysis was done using R (version 3.6), Seurat (4.3.0) Harmony R (v3)

For manuscripts utilizing custom algorithms or software that are central to the research but not yet described in published literature, software must be made available to editors and reviewers. We strongly encourage code deposition in a community repository (e.g. GitHub). See the Nature Portfolio guidelines for submitting code & software for further information.

Data

Policy information about availability of data

All manuscripts must include a data availability statement. This statement should provide the following information, where applicable:

- Accession codes, unique identifiers, or web links for publicly available datasets
- A description of any restrictions on data availability
- For clinical datasets or third party data, please ensure that the statement adheres to our policy

The data that support the findings in this study are available within the article and its Supplementary Information files, and from the corresponding author upon request. Raw scRNAseq data generated in this study has been deposited at the GEO database under accession code: GSE221500; https://www.ncbi.nlm.nih.gov/geo/query/acc.cgi?acc=GSE221500.

Research involving human participants, their data, or biological material

Policy information about studies with human participants or human data. See also policy information about sex, gender (identity/presentation), and sexual orientation and race, ethnicity and racism.

Reporting on sex and gender	Not applicable
Reporting on race, ethnicity, or other socially relevant groupings	Not applicable
Population characteristics	Not applicable
Recruitment	Not applicable
Ethics oversight	Not applicable

Note that full information on the approval of the study protocol must also be provided in the manuscript.

Field-specific reporting

Please select the one below that is the best fit for your research. If you are not sure, read the appropriate sections before making your selection.

☒ Life sciences ☐ Behavioural & social sciences ☐ Ecological, evolutionary & environmental sciences

For a reference copy of the document with all sections, see nature.com/documents/nr-reporting-summary-flat.pdf

Life sciences study design

All studies must disclose on these points even when the disclosure is negative.

Sample size	No sample size calculation was performed in in vitro experiment. We chose the corresponding sample size based on the literature which was sufficient to analyze significance between groups.
Data exclusions	No samples were excluded from the analysis.
Replication	All data were generated from more than 3 independent experiments and number of reproductions for each experimental finding is described in each figure legend.
Randomization	N/A
Blinding	For RNA sequencing, blinding was not necessary because data was analyzed based on unbiased clustering. The design of other experiments did not require blinding as the outcome was not dependent on the judgement of the investigator and could not be influenced by prior knowledge of the groups.

Reporting for specific materials, systems and methods

We require information from authors about some types of materials, experimental systems and methods used in many studies. Here, indicate whether each material, system or method listed is relevant to your study. If you are not sure if a list item applies to your research, read the appropriate section before selecting a response.

Materials & experimental systems

n/a	Involved in the study	
☐	☒	Antibodies
☐	☒	Eukaryotic cell lines
☒	☐	Palaeontology and archaeology
☒	☐	Animals and other organisms
☒	☐	Clinical data
☒	☐	Dual use research of concern
☒	☐	Plants

Methods

n/a	Involved in the study	
☒	☐	ChIP-seq
☐	☒	Flow cytometry
☒	☐	MRI-based neuroimaging

Antibodies

Antibodies used

anti-SIRPa-PeCy7 (Biolegend#323807,clone SE5A5) anti-cardiac isoform of cTNT (ThermoFisher Scientific #MA5--12960, clone 13-11), anti-myosin light chain 2 (Abcam #79935 polyclonal) anti-CD90-APC (BD PharMingen #559869, clone 5E10), anti-FN1 (Abcam #2413), anti-COL1 (Abcam #34710), anti-COL3 (Abcam #7778), anti-CD9-FITC (Abcam #18241), anti-MYH11 (Abcam #133567), anti-WT1 (Abcam #89901), anti-GFP (Rockland #60010215), Cx43 (Abcam #11370)
goat anti-mouse IgG-APC (BD PharMingen #550826, polyclonal), donkey anti-rabbit IgG-PE (Jackson ImmunoResearch #711-116-152, polyclonal), donkey anti-mouse IgG-Alexa488 (ThermoFisher #R37114, polyclonal), donkey anti-rabbit IgG-Alexa555 (ThermoFisher #31572, polyclonal), donkey anti-rabbit IgG-Alexa488 (ThermoFisher #R37118, polyclonal), donkey anti-mouse IgG-Alexa555 (ThermoFisher #A31570, polyclonal) Aldefluor kit (Stem Cell Technologies #10700)

Validation

Validation from supplier website. Antibodies were validated to react with human proteins by flow cytometric analysis.
anti-SIRPA-PeCy7, anti-CD90-APC, CD9-FITC

Validation from supplier website. Antibodies were validated to react with human proteins by immunohistochemical analysis
rabbit anti-human cTNT, rabbit anti-CX43, anti-FN1, anti-COL1, anti-COL3, anti-GFP, anti-WT1, anti-MYH11

Eukaryotic cell lines

Policy information about cell lines and Sex and Gender in Research

Cell line source(s)

HES2 (WiCell) , HES2-RFP/GFP (Irion et al., 2007), human cardiac fibroblast (Lonza, #CC-2904; Promocell, #C-14036)

Authentication

Cells were authentificated based on their morphology, growth condition and specific gene expression.

Mycoplasma contamination

Cell lines were tested negative for mycoplasma contamination.

Commonly misidentified lines
(See ICLAC register)

No commonly misidentified cell lines were used in the study.

Plants

Seed stocks

Not applicable

Novel plant genotypes

Not applicable

Authentication

Not applicable

Flow Cytometry

Plots

Confirm that:

- [x] The axis labels state the marker and fluorochrome used (e.g. CD4-FITC).
- [x] The axis scales are clearly visible. Include numbers along axes only for bottom left plot of group (a 'group' is an analysis of identical markers).
- [x] All plots are contour plots with outliers or pseudocolor plots.
- [x] A numerical value for number of cells or percentage (with statistics) is provided.

Methodology

Sample preparation	The EBs were dissociated by incubation in Collagenase type 2 (0.5mg/ml) in HANKs buffer overnight at room temperature followed by TrypLE for 5 mins at 37 C. For cell-surface marker analyses, cells were stained for 30 min at 4 C in FACS buffer consisting of PBS with 5% fetal calf serum (FCS) and 0.02% sodium azide. For intracellular staining, cells were fixed for 20mins at 4 C with 4% PFA in PBS followed by permeabilization using 90% methanol for 20 mins at 4 C. Cells were washed with PBS containing 5% FCS and stained with unconjugated primary antibodies in FACS buffer overnight at 4 C. Stained cells were washed with PBS with 5% FCS and stained with secondary antibodies in FACS buffer for 30 mins at 4 C.
Instrument	LSR II Flow cytometer or LSRFortessa (BD) was used for analysis. For the sample preparation for scRNAseq, DAPI(-) live cells were sorted using FACSAriaRITT (BD).
Software	FACS DIVA was used for data collection, and FlowJo v10 was used for data analysis.
Cell population abundance	100,000
Gating strategy	Cells were first gated based on light scatter properties based on cell size (FSC-A) and granularity (SSC-A), and width parameter<br>on forward scatter was used to gate out doublets. Negative cells were gated based on secondary only staining control. Cells stained with SIRPA, CD90, CD9 or ALDH were classified based on unstained negative cells.

- [x] Tick this box to confirm that a figure exemplifying the gating strategy is provided in the Supplementary Information.
